# Transcatheter aortic valve implantation-related acute ascending aortic dissection diagnosed intraoperatively using transoesophageal echocardiography: a case series

**DOI:** 10.1093/ehjcr/ytag457

**Published:** 2026-06-16

**Authors:** Yuki Makizawa, Atsushi Fujiwara, Takanobu Fujisawa, Yusuke Kusaka, Toshiaki Minami

**Affiliations:** Department of Anaesthesiology, Osaka Medical and Pharmaceutical University, Takatsuki, Osaka 569-8686, Japan; Department of Anaesthesiology, Osaka Medical and Pharmaceutical University, Takatsuki, Osaka 569-8686, Japan; Department of Anaesthesiology, Osaka Medical and Pharmaceutical University, Takatsuki, Osaka 569-8686, Japan; Department of Anaesthesiology, Osaka Medical and Pharmaceutical University, Takatsuki, Osaka 569-8686, Japan; Department of Anaesthesiology, Osaka Medical and Pharmaceutical University, Takatsuki, Osaka 569-8686, Japan

**Keywords:** Transcatheter aortic valve implantation, Acute ascending aortic dissection, Transoesophageal echocardiography, Intraoperative diagnosis, Surgical conversion, Case series

## Abstract

**Background:**

Transcatheter aortic valve implantation (TAVI) has been widely adopted as a less invasive treatment for aortic stenosis (AS). Although rare, acute ascending aortic dissection associated with TAVI is a life-threatening complication that necessitates prompt intraoperative diagnosis. This case series reports two cases of acute ascending aortic dissection occurring during TAVI performed under general anaesthesia.

**Case summary:**

Cases 1 and 2 involved an 87-year-old woman and a 90-year-old man, respectively, both of whom underwent transfemoral TAVI for severe AS. In both cases, procedural difficulty was encountered, including valve pop-up and resistance during device passage. Before overt haemodynamic deterioration became apparent, intraoperative transoesophageal echocardiography revealed an intimal flap and false lumen in the ascending aorta, leading to the diagnosis of acute ascending aortic dissection. Both patients underwent prompt surgical intervention. Case 1 underwent surgical aortic valve replacement (SAVR) and ascending aortic replacement, whereas Case 2 underwent SAVR and total arch replacement. Both patients were discharged ambulatory without neurological sequelae.

**Discussion:**

Unlike fluoroscopy and angiography, which provide only intermittent assessment, transoesophageal echocardiography enables continuous intraoperative evaluation and can simultaneously detect findings directly relevant to haemodynamic status, including an intimal flap, false lumen, acute aortic regurgitation, and pericardial effusion. This case series suggests that additional transoesophageal echocardiography assessment prompted by procedural difficulty may contribute to the early diagnosis of serious complications and facilitate prompt conversion to surgical intervention.

Learning points

- Acute ascending aortic dissection should be suspected during transcatheter aortic valve implantation (TAVI) when procedural difficulty occurs, even without marked haemodynamic instability.
- Intraoperative transoesophageal echocardiography can detect both direct and haemodynamically significant findings of acute aortic dissection during TAVI.
- Additional echocardiographic assessment triggered by procedural difficulty may facilitate early diagnosis and prompt surgical intervention.

## Introduction

Transcatheter aortic valve implantation (TAVI) has been widely adopted as a less invasive treatment for aortic stenosis (AS) compared to surgical aortic valve replacement (SAVR). Current guidelines recommend that the treatment strategy should be determined by the multidisciplinary cardiovascular team based on patient characteristics and anatomical considerations.^1^ However, acute ascending aortic dissection has been reported as a serious complication of TAVI.^2^

## Summary figure

**Figure:**
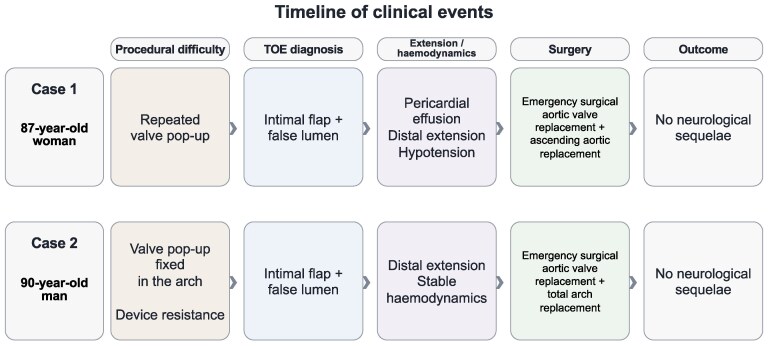


In recent years, transfemoral TAVI has increasingly been performed under local anaesthesia with sedation. However, at our institution, intraoperative transoesophageal echocardiography (TOE) is routinely used, and all TAVI procedures are thus performed under general anaesthesia.

In the present report, we described two cases of acute ascending aortic dissection that occurred during TAVI. In both cases, dissection was diagnosed via intraoperative TOE before overt haemodynamic deterioration became apparent, allowing prompt conversion to surgical repair.

## Patient 1

An 87-year-old woman (height 143 cm, weight 46 kg) under follow-up for AS developed chest pain, for which TAVI under general anaesthesia was planned. Her medical history was significant for hypertension. Blood tests revealed no anaemia or renal dysfunction. A 12-lead electrocardiogram indicated sinus rhythm without significant ST-segment changes. Transthoracic echocardiography (TTE) demonstrated severe AS, with a peak transvalvular velocity of 3.7 m/s, a mean pressure gradient of 36 mmHg, a peak pressure gradient of 55.1 mmHg, and an aortic valve area of 0.83 cm^2^ calculated using the continuity equation. Left ventricular hypertrophy was present, with a left ventricular ejection fraction of 71%, a left ventricular end-diastolic diameter of 44 mm, and a left ventricular end-systolic diameter of 26 mm. The aortic valve was tricuspid with severe calcification, and mild aortic regurgitation was observed. Non-contrast computed tomography revealed an annular perimeter of 68.1 mm. The aortic valve calcium score was 163 Agatston units. Preprocedural coronary angiography revealed 75% stenosis in segment 13 of the left circumflex artery; however, no percutaneous coronary intervention was performed before TAVI.

**Figure 1:**
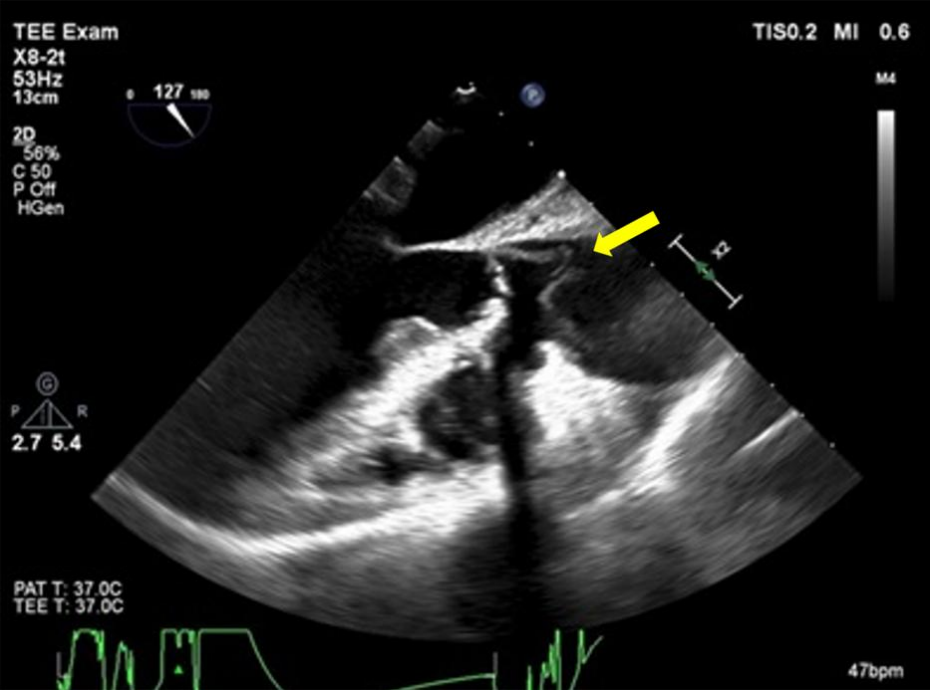
Intraoperative transoesophageal echocardiography in Case 1 (mid-oesophageal aortic valve long-axis view) demonstrates an intimal flap (arrow) and false lumen in the ascending aorta.

**Figure 2:**
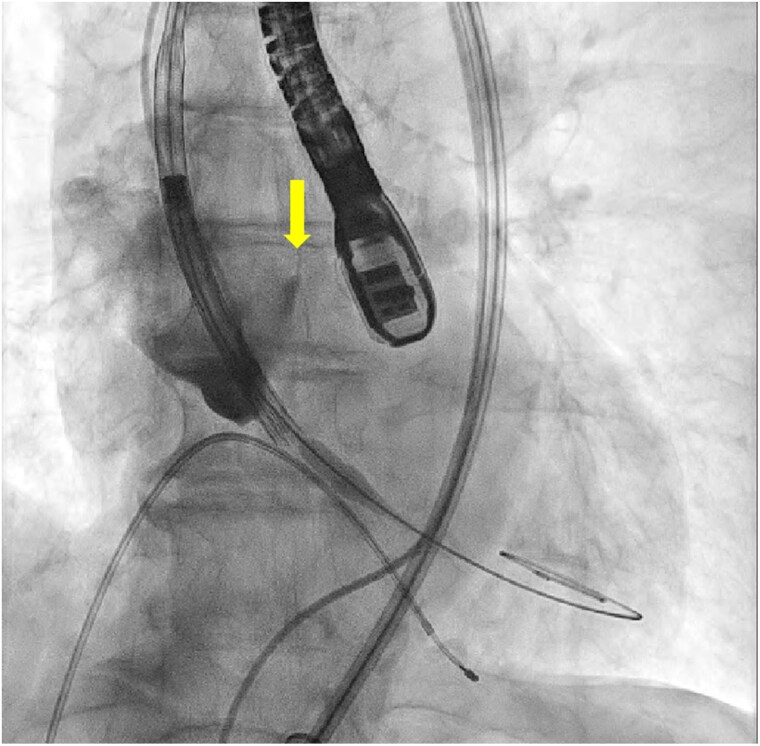
Aortography in Case 1 depicts poor contrast opacification of the left sinus of Valsalva, as indicated by the arrow, after interruption of the transcatheter aortic valve implantation procedure.

Following cardiac team discussion, transfemoral TAVI using an Evolut PRO+ 26 mm valve (Medtronic, Minneapolis, MN, USA) was planned. Anaesthetic induction was uneventful. In addition to standard monitoring, a left radial arterial line and a central venous catheter via the right internal jugular vein were placed. After balloon aortic valvuloplasty, valve deployment was attempted twice during controlled pacing at 100 beats/min; however, the valve dislodged upward (pop-up) on both occasions. A third attempt during pacing at 120 beats/min again resulted in valve pop-up. Real-time TOE demonstrated an intimal flap and false lumen in the ascending aorta (*Figure 1*, Supplementary material online, *Video S1*). The procedure was therefore interrupted, and aortography revealed poor contrast opacification of the left sinus of Valsalva (*Figure 2*, Supplementary material online, *Video S2*). TOE subsequently revealed pericardial effusion and extension of the dissection into the descending aorta, and emergency conversion to SAVR and ascending aortic replacement was planned. Until pericardial effusion was detected, systolic blood pressure had remained in the 90–100 mmHg range, but thereafter decreased to the 60–70 mmHg range, requiring a continuous noradrenaline infusion at 0.1 μg/kg/min. No electrocardiographic changes or worsening of aortic regurgitation were observed.

Following median sternotomy, cardiopulmonary bypass was established with right axillary artery cannulation for arterial inflow and two-stage right atrial cannulation for venous drainage. Intraoperatively, an approximately 3-cm entry tear was identified just above the sino-tubular junction over the non-coronary sinus (see Supplementary material online, *Video S3*). Surgical bioprosthetic aortic valve replacement and ascending aortic graft replacement were performed. Weaning from cardiopulmonary bypass was uneventful and required only minimal vasopressor support. The patient was admitted to the intensive care unit, extubated on postoperative day 2, and discharged from the intensive care unit on postoperative day 3. She was discharged ambulatory on postoperative day 26 without neurological sequelae.

## Patient 2

A 90-year-old man (height 157 cm, weight 44 kg) had a history of exertional dyspnoea for 4 years and had been managed conservatively. However, TAVI under general anaesthesia was planned owing to worsening symptoms. His medical history included hypertension, previous right nephrectomy, and myocardial infarction treated with percutaneous coronary intervention 21 years earlier. Preprocedural coronary angiography showed no significant coronary stenosis. Blood tests revealed no evidence of anaemia. Renal dysfunction was noted, with a creatinine level of 2.04 mg/dL and an estimated glomerular filtration rate of 25 mL/min/1.73 m^2^. A 12-lead electrocardiogram showed sinus rhythm without significant ST-segment changes. TTE demonstrated severe AS, with a peak transvalvular velocity of 4.6 m/s, mean pressure gradient of 55 mmHg, peak pressure gradient of 82.8 mmHg, and aortic valve area of 0.99 cm^2^ calculated using the continuity equation. Left ventricular hypertrophy was present, with a left ventricular ejection fraction of 53%, left ventricular end-diastolic diameter of 50 mm, and left ventricular end-systolic diameter of 36 mm. The aortic valve was tricuspid with severe calcification, and moderate aortic regurgitation was present. Non-contrast computed tomography showed an annular perimeter of 68.6 mm. The aortic valve calcium score was 4326 Agatston units.

**Figure 3:**
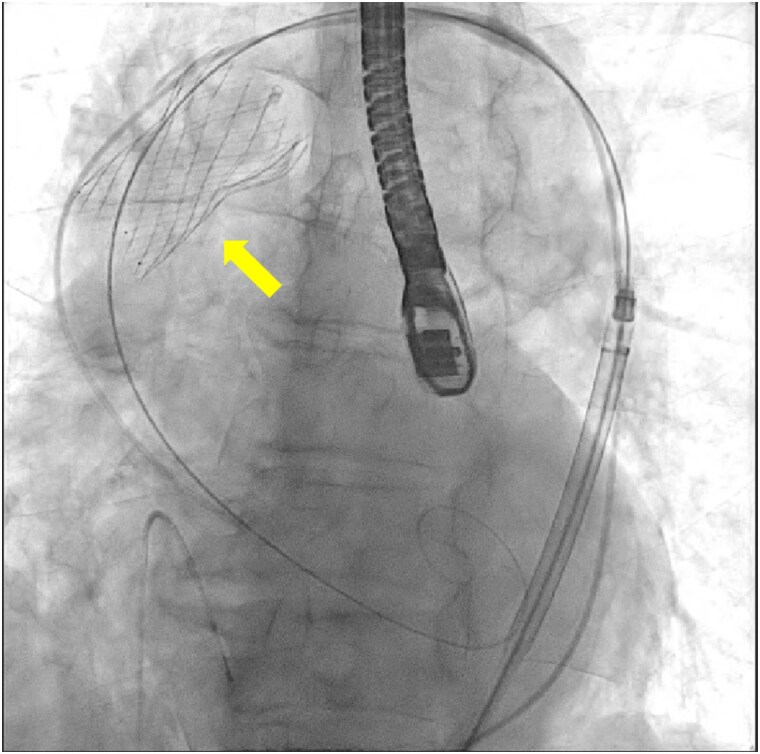
Fluoroscopic image in Case 2 demonstrates a pop-up of the first self-expanding valve, which became fixed in the aortic arch (arrow).

**Figure 4:**
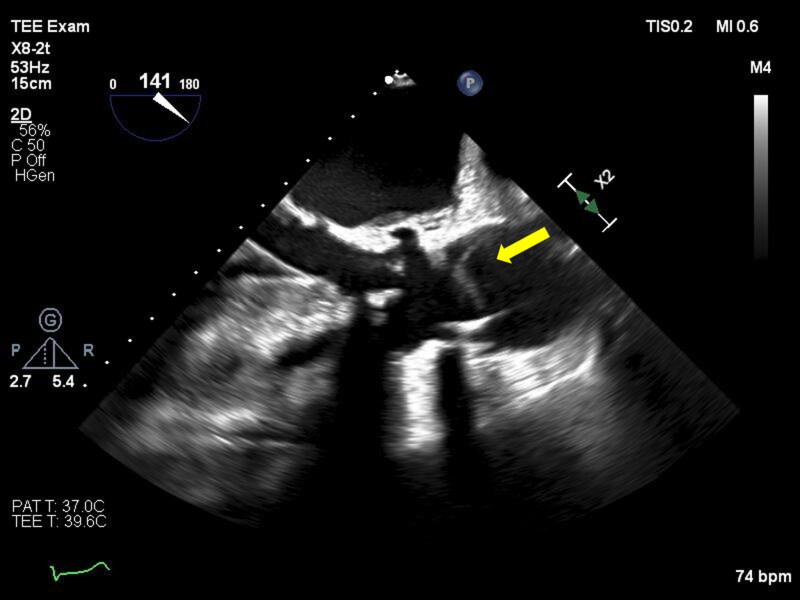
Intraoperative transoesophageal echocardiography in Case 2 (mid-oesophageal aortic valve long-axis view) demonstrates a newly developed intimal flap (arrow) and false lumen in the ascending aorta immediately after deployment of the second valve.

**Figure 5:**
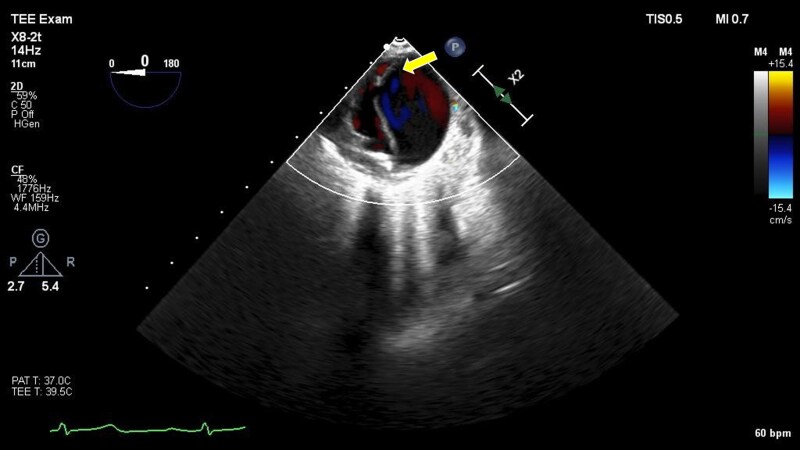
Intraoperative transoesophageal echocardiography demonstrates an intimal flap in the descending aorta (arrow).

After cardiac team discussion, transfemoral TAVI using an Evolut FX 26 mm valve (Medtronic, Minneapolis, MN, USA) was planned. Anaesthetic induction was uneventful. In addition to standard monitoring, a left radial arterial line and a central venous catheter via the right internal jugular vein were placed. After balloon aortic valvuloplasty, valve deployment was performed during rapid pacing at 180 beats/min; however, the valve popped up immediately and became fixed in the aortic arch (*Figure 3*). At that point, there was no apparent hypotension or other finding suggestive of aortic dissection. Before retrieval of the dislodged valve, implantation of a second valve was planned. Mild resistance was encountered while advancing the device through the lumen of the first valve, although deployment was achieved. Immediately after deployment of the second valve, TOE revealed a newly developed intimal flap and false lumen in the ascending aorta (*Figure 4*, Supplementary material online, *Video S4*). At this juncture, there was still no evidence of hypotension, pericardial effusion, electrocardiographic change, or worsening of aortic regurgitation. However, TOE confirmed extension of the dissection into the descending aorta (*Figure 5*), and emergency conversion to SAVR and total arch replacement was decided.

Following median sternotomy, cardiopulmonary bypass was established with right axillary artery cannulation for arterial inflow and two-stage right atrial cannulation for venous drainage. Intraoperatively, an approximately 1-cm entry tear was identified on the greater curvature of the ascending aorta, approximately 3 cm distal to the sino-tubular junction. Surgical bioprosthetic aortic valve replacement and total arch replacement were performed. Weaning from cardiopulmonary bypass was uneventful, requiring only minimal vasopressor support. The patient was admitted to the intensive care unit, extubated on postoperative day 2, and discharged from the intensive care unit on postoperative day 7. He developed pneumonia after extubation, which required additional postoperative treatment and prolonged his hospital stay. He was discharged ambulatory on postoperative day 50 without neurological sequelae.

## Discussion

TAVI-related aortic dissection is rare but life-threatening; therefore, it is essential to identify clues that may raise suspicion and lead to diagnosis.^2–4^ From the initiation of our institutional TAVI programme to the second case, 252 TAVI procedures were performed, and the present cases occurred as the 138th and 252nd procedures. In both present cases, acute aortic dissection was diagnosed via TOE after procedural events, such as valve pop-up or difficulty during device manipulation, were encountered, before overt haemodynamic deterioration became apparent, allowing prompt conversion to surgical repair. In such settings, where obvious symptoms or haemodynamic instability may be absent, diagnosis may otherwise be delayed.^4^

During TAVI, in addition to echocardiography, fluoroscopy and aortography are commonly used to determine valve position and assess procedural complications.^5^ However, contrast angiography is usually performed only at specific procedural time points, such as before or after valve deployment, making it unsuitable for continuous intraprocedural assessment.^5^ In contrast, TOE allows continuous intraoperative monitoring and can simultaneously evaluate direct signs of dissection, such as an intimal flap and false lumen, and haemodynamically relevant findings, including acute aortic regurgitation, pericardial effusion, and regional wall motion abnormalities.^5,6^ Nevertheless, TTE may be limited by image quality in the supine position, restricted visualization of the aortic root and ascending aorta, and interference from fluoroscopic equipment, making continuous assessment difficult.^6,7^

In recent years, a minimalist approach using local anaesthesia with sedation has increasingly been adopted for transfemoral TAVI, which is associated with fewer respiratory complications and shorter hospital stay than approaches involving general anaesthesia.^8,9^ Comparative studies have demonstrated similar 30-day major composite outcomes between minimalist and general anaesthesia approaches, although the reported association with shorter length of stay should be interpreted cautiously owing to potential confounding.^9,10^ Even when TAVI is initiated using a minimalist approach, conversion to general anaesthesia may be required because of procedural complications or patient movement, and the use of TOE may be limited under sedation in some patients.^11^ Thus, the choice of anaesthetic strategy may influence the feasibility and role of intraoperative monitoring, including TOE.

Overall, our results suggest that intraoperative TOE may be particularly useful in selected TAVI cases in which technical difficulty or a higher risk of procedural complications is anticipated. In such situations, TOE may provide complementary continuous assessment that is difficult to achieve with fluoroscopy or angiography alone, while also allowing integrated evaluation of haemodynamically relevant findings. In our two cases, additional TOE assessment prompted by procedural difficulty contributed to the diagnosis of acute aortic dissection and facilitated early conversion to surgical repair.

## Supplementary Material

ytag457_Supplementary_Data

## Data Availability

The data underlying this article are available in the article.

## Lead author biography

Dr Yuki Makizawa graduated from Osaka Medical and Pharmaceutical University in 2017 and currently works at Osaka Medical and Pharmaceutical University, Japan. His clinical interests include perioperative anaesthesia, particularly cardiac anaesthesia.

## Notes

**Consent:** Written informed consent for publication of this case series and accompanying images was obtained from all patients included in this report.
